# Asymmetric Wavefront Aberrations and Pupillary Shapes Induced by Electrical Stimulation of Ciliary Nerve in Cats Measured with Compact Wavefront Aberrometer

**DOI:** 10.1371/journal.pone.0105615

**Published:** 2014-08-21

**Authors:** Suguru Miyagawa, Toshifumi Mihashi, Hiroyuki Kanda, Yoko Hirohara, Takao Endo, Takeshi Morimoto, Tomomitsu Miyoshi, Takashi Fujikado

**Affiliations:** 1 Department of Applied Visual Science, Osaka University Graduate School of Medicine, Suita, Osaka, Japan; 2 Topcon Corporation Research Institute, Itabashi, Tokyo, Japan; 3 Innovative Research Initiatives, Tokyo Institute of Technology, Yokohama, Kanagawa, Japan; 4 Department of Opthalmology, Osaka University Graduate School of Medicine, Suita, Osaka, Japan; 5 Department of Integrative Physiology, Osaka University Graduate School of Medicine, Suita, Osaka, Japan; Charité University Medicine Berlin, Germany

## Abstract

To investigate the changes in the wavefront aberrations and pupillary shape in response to electrical stimulation of the branches of the ciliary nerves in cats. Seven eyes of seven cats were studied under general anesthesia. Trains of monophasic pulses (current, 0.1 to 1.0 mA; duration, 0.5 ms/phase; frequency, 5 to 40 Hz) were applied to the lateral or medial branch of the short ciliary nerve near the posterior pole of the eye. A pair of electrodes was hooked onto one or both branch of the short ciliary nerve. The electrodes were placed about 5 mm from the scleral surface. The wavefront aberrations were recorded continuously for 2 seconds before, 8 seconds during, and for 20 seconds after the electrical stimulation. The pupillary images were simultaneously recorded during the stimulation period. Both the wavefront aberrations and the pupillary images were obtained 10 times/sec with a custom-built wavefront aberrometer. The maximum accommodative amplitude was 1.19 diopters (D) produced by electrical stimulation of the short ciliary nerves. The latency of the accommodative changes was very short, and the accommodative level gradually increased up to 4 seconds and reached a plateau. When only one branch of the ciliary nerve was stimulated, the pupil dilated asymmetrically, and the oblique astigmatism and one of the asymmetrical wavefront terms was also altered. Our results showed that the wavefront aberrations and pupillary dilations can be measured simultaneously and serially with a compact wavefront aberrometer. The asymmetric pupil dilation and asymmetric changes of the wavefront aberrations suggest that each branch of the ciliary nerve innervates specific segments of the ciliary muscle and dilator muscle of the pupil.

## Introduction

Lens accommodation and pupillary dilation or constriction elicited by electrical stimulation of the peripheral nerves or the brain have been extensively studied. The changes in the refractive power of the eye, i.e., accommodation, to electrical stimulation of the ciliary ganglion have been studied in cats and other animals [1]–[3]. The amplitude of accommodation was dependent on the frequency and voltage of the electrical stimulus. The maximum amplitude of accommodation was about 2 diopters. The accommodative responses elicited by microstimulation of the midbrain or cerebellum have also been studied in cats [4]–[6]. Glasser et al. demonstrated the accommodative responses elicited by stimulation of the preganglionic Edinger-Westphal nucleus in rhesus monkeys [7], [8]. In monkeys, the maximum amplitude of accommodation was 10 to 20 diopters.

The changes in the accommodation not only affected the refractive power but also the ocular aberrations. An ideal monochromatic ray of light from a point source has a perfect spherical wave-front surface. If the eye has an ocular aberration, the wavefront of light reflected from the ocular fundus deviate from an ideal spherical surface, and the deviation in the wavefront is called the wavefront aberrations [9]. Many studies have demonstrated changes in the wavefront aberrations induced by lens accommodation using different techniques in humans. Atchison et al. studied the aberrations with the Howland aberroscope technique [10], and He et al. applied psychophysical ray tracing methods [11]. Recently, Shack-Hartmann wavefront aberrometer (SHWA) techniques allowed the rapid and accurate measurements of the wavefront aberrations [12]–[14]. With a SHWA, changes of the Zernike terms, e.g., astigmatism, coma, and spherical aberration, can be evaluated during and after accommodative changes. Some studies have demonstrated a significant increase of negative spherical aberration during accommodation using SHWA [15], [16]. Animal models have also been used to evaluate changes in the wavefront aberrations with SHWA. For example, Huxlin et al. measured up to sixth order wavefront aberrations in wake cats [17]. Ramamirtham et al. also measured the wavefront aberrations in young monkeys [18].

The dynamic pupillary dilation and constriction evoked by light stimulation or by electrical stimulation of the ciliary nerve have also been studied in cats [19], [20]. In addition, the dynamic pupillary dilations and eye movements in response to microstimulation of the superior colliculus or the optic tectum have been studied in monkeys and birds [21], [22]. Dearworth et al. studied the pupillary constriction evoked in vitro by stimulating the ciliary nerve in turtles [23].

Clinically, patients with Adie’s syndrome have tonically dilated pupils and accommodative palsy. In addition, the pupillary reactions in these patients are usually segmental due to sector iridoplegia. Bell and Thompson reported that astigmatism was induced with accommodation in one-third of Adie’s patients, and they suggested that this may be related to the segmental paralysis of the ciliary muscle [24].

Because both lens accommodation and pupillary constriction and dilation are controlled by postganglionic nerve fibers travelling in the short ciliary nerves, measurements of pupillary diameter and circularity, as well as lens accommodation and wavefront abberrations should be effected by electrical stimulation of the short ciliary nerves. In cats, the short ciliary nerve is made up of a lateral and a medial branch, so segmental stimulation is possible.

The purpose of this study was to determine the dynamic changes in the accommodation, wavefront aberrations, and pupillary size and shape evoked by electrical stimulation of one or both branches of the short ciliary nerve near the posterior pole of the eye in cats. The responses were measured with a custom-made, compact SHWA (Topcon Corporation and Aston University) which allowed us to determine the dynamic changes of accommodation, wavefront aberrations, pupillary size and shape simultaneously [25].

## Materials and Methods

### Experimental Animals

Seven healthy adult cats between 10- to 14 months-of-age were studied. These cats were raised in a breeding colony in the Institute of laboratory Animals, Osaka University, Graduate School of Medicine. The cats were initially injected with atropine sulfate (0.1 mg/kg) intraperitoneally and after 30 minutes they were anesthetized with an intramuscular injection of ketamine hydrochloride (25 mg/kg). The anesthesia was maintained by a continuous intravenous infusion of pentobarbital sodium (1 mg/kg/hr). The cats were paralyzed by an infusion of pancuronium bromide (0.2 mg/kg/hr) mixed with Ringer’s solution and glucose (0.1 g/kg/hr), and artificially ventilated with equivalent mixture of nitrous oxide (N^2^O) and oxygen (O^2^) for auxiliary anesthesia and alleviation of pain.

The end-tidal CO^2^ concentration was controlled at 3.5 to 5.0% by altering the frequency and tidal volume of ventilation. The intratracheal pressure and electrocardiogram were also monitored, and the body temperature was maintained at 38°C with a heating pad. The cornea of cats was kept moist using custom made contact lenses during the experiments.

This study was carried out in strict accordance with the recommendations in the Guide for the Care and Use of Animals of the National Institutes of Health. The procedures were approved by the Animal Research Committee of the Osaka University Medical School, document number 20–145. All surgery was performed under pentobarbital sodium anesthesia, and all efforts were made to minimize pain. All animals were sacrificed by a rapid intravenous infusion of pentobarbital sodium (64.8 mg/ml) after a completion of all experimental procedures.

### Electric Stimulation of Ciliary Nerve

**Figure 1:**
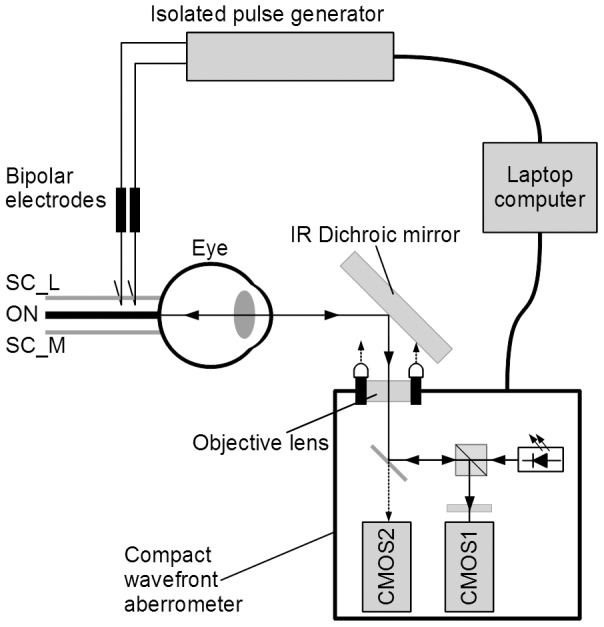
Photograph of the compact wavefront aberrometer attached to a flex holder arm.

In cats, the ciliary ganglion gives rise to a lateral and a medial branch of the short ciliary nerve. Additionally, one or two fine communicating branches from the long ciliary nerve are fused with the short ciliary nerve. Electrical currents were applied to either the lateral or medial branch or to both branches of the short ciliary nerves to study the changes in the accommodation, pupillary size and shape, and wavefront aberrations. A schematic diagram of the experimental setup is shown in Figure 1. The stimulating electrodes were bipolar hook-shaped electrodes made of 0.3 mm diameter stainless steel wire (OK212-069, Unique Medical, Tokyo, Japan). The wire was coated with insulating resin with a small region of the tip bared where the electrode contacted the ciliary nerve. The electrodes were hooked onto the branch of the short ciliary nerve about 5 mm from sclera. Trains of monophasic square wave electrical pulses were applied to either the lateral or the medial or to both branches of the short ciliary nerve. All pulses were generated by an isolated pulse generator (STG2008, Multi Channel Systems MCS GmbH, Reutlingen Germany). The pulse parameters were: current intensities of 0.1, 0.3, 0.5, and 1.0 mA; frequencies of 5, 10, 20, and 40 Hz; pulse duration of 0.5 ms/phase; and the duration of the pulse trains was 8 s. Changes in the accommodation, wavefront aberrations and pupillary shape evoked by electrical stimulation of short ciliary nerve were simultaneously recorded with a compact wavefront aberrometer.

### Dynamic Measurements of Wavefront Aberrations Using Compact Wavefront Aberrometer

**Figure 2:**
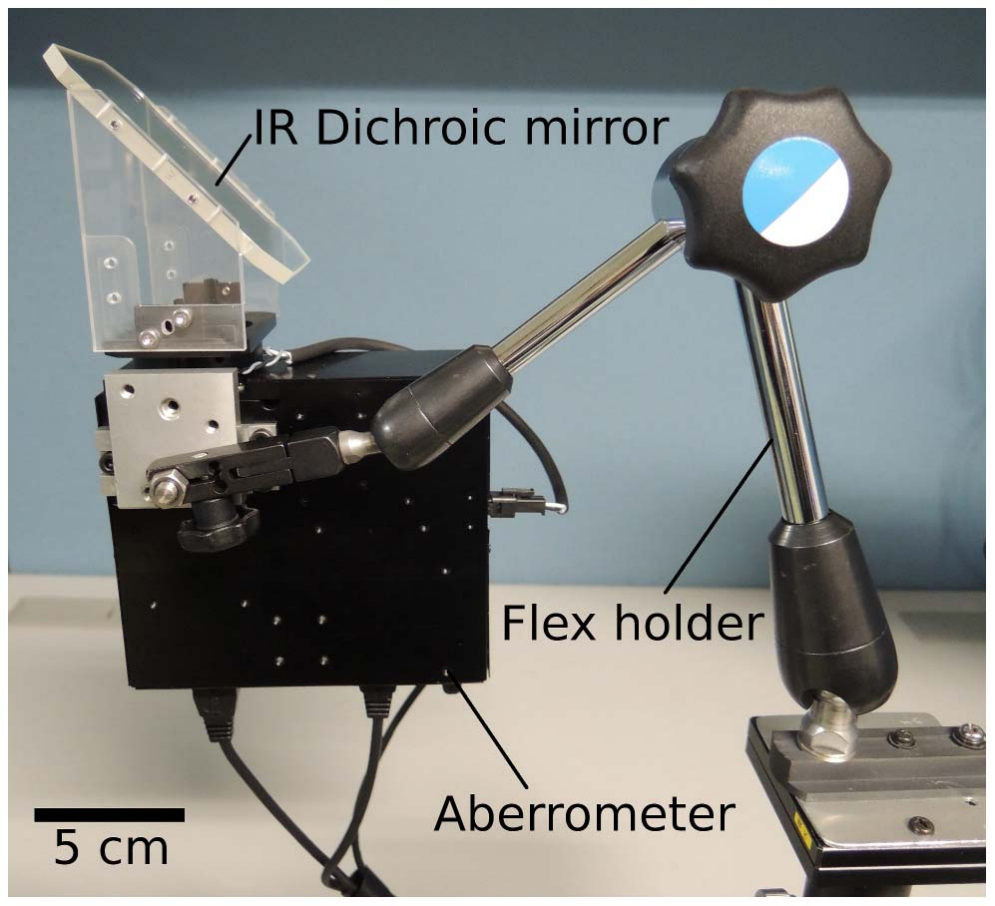
Schematic diagram of experimental setup. ON, optic nerve; SC_L, lateral branch of the short ciliary nerve; SC_M, medial branch of the short ciliary nerve. CMOS1, complementary metal-oxide semiconductor (CMOS) image sensor for Shack-Hartmann spot image; CMOS2, CMOS image sensor for anterior eye (pupil) image.

The wavefront aberrations were measured with a compact wavefront aberrometer. This device consisted of an open-field dichroic mirror and a cuboid shaped body (12×12×4.5 cm), which can be attached to a standard flex holder (Figure 1). Because of the flexibility and compactness of this aberrometer, it can be easily used for *in vivo* animal studies or clinical studies. The experimental setup and optical arrangement are shown in Figure 2. The aberrometer contained two complementary metal-oxide semiconductor (CMOS) image sensors. The wavefront aberrations were measured with the first CMOS sensor (CMOS1). A lenslet array plate located in front of CMOS1 focused the Shack-Hartmann spot images. The digitized Shack-Hartmann spot images were recorded sequentially at 10 frames/sec. Therefore, we were able to measure the changes of accommodation and aberrations every 100 ms. The digitized Shack-Hartmann spot images were analyzed quantitatively for up to the 6th order by expanding the set of Zernike polynomials with custom written software.

The second CMOS sensor (CMOS2) obtained the images of the anterior segment of the eye to evaluate the pupillary shape. Both the aberrometer and the isolated pulse generator were synchronously controlled by a commercially available laptop computer.

**Figure 3:**
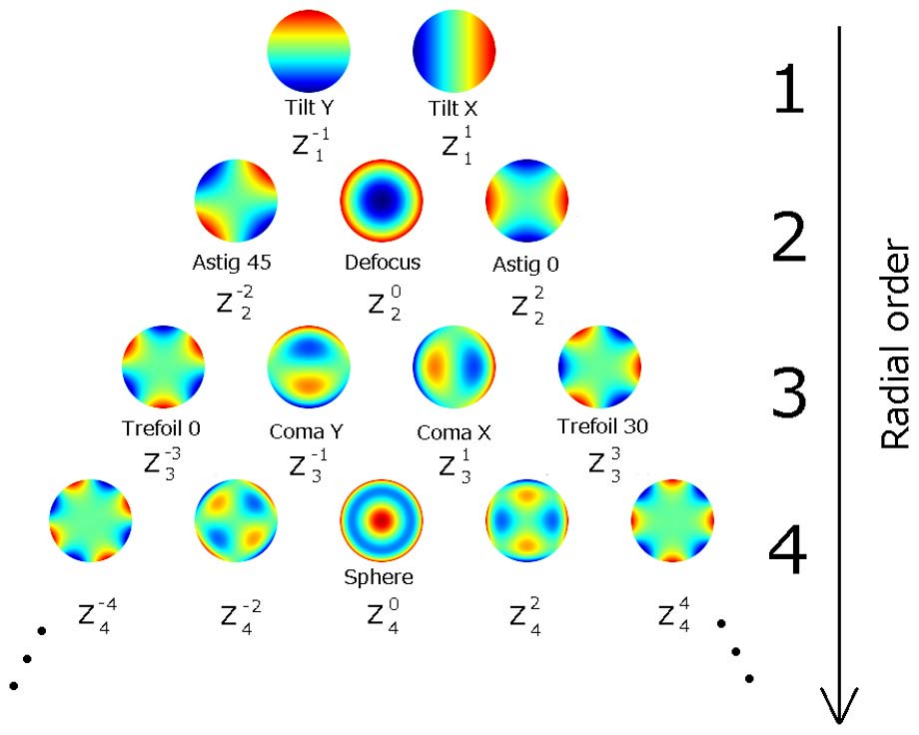
Color map diagram of Zernike polynomials up to the 4th order. Astig 45, oblique astigmatism (45 deg); Astig 0, with-the-rule astigmatism (0 deg); Trefoil 0, vertical trefoil aberration (0 deg); Trefoil 30, oblique trefoil aberration (30 deg); Sphere, spherical aberration.

The accommodative responses were assessed by the changes of the refractive power (spherical equivalents). The wavefront aberrations were evaluated by the changes of the Zernike coefficients, and the wavefront aberrations were specified using the standard nomenclature defined with reference to the standard coordinate system recommended by the Optical Society of America [26]. A color map diagram of Zernike polynomials of up to 4th order is shown in Figure 3. The with- and against-the-rule astigmatism (Z^2^_2_), the oblique astigmatism (Z^−2^_2_), the trefoil terms (Z^−3^_3_ and Z^3^_3_), the x coma (Z^1^_3_), the y coma (Z^−1^_3_), and spherical aberration term (Z^0^_4_) were determined.

To study the dynamic changes of accommodation, we determined the velocity of the accommodative responses where the velocity was defined in diopters/sec and we assessed the maximum amplitude of accommodation and the time required to reach 80% of the maximum amplitude. Then the velocity ratio was calculated as 80% of maximum amplitude divided by the time to reach this level, i.e., 0.8 amplitude of accommodation in diopters/time in msec. In cases where the wavefront aberration was not measureable due to pupillary constriction, the pupil was dilated by 5% phenylephrine HCl (Neosynesin). Earlier studies showed that 10% neosynephrine eye drops did not alter the accommodation in cats [27].

### Measurements of Pupillary Shape

To study pupillary dilation and constriction, the pupil was photographed by CMOS image sensor incorporated into the wavefront aberrometer. The images were analyzed with a custom written software. The contour of the pupil was first outlined to calculate the area of the pupil (mm^2^) and to evaluate the pupil shape. The coordinates of the center of gravity of the pupil were also calculated to determine whether there was an asymmetrical change in the pupillary shape. This program can determine both the size and shape of the pupil. To study the relationship between the changes in the wavefront aberrations and pupillary shape, both were recorded simultaneously and sequentially at 10 frames/sec for 2 seconds before, 8 seconds during, and for 20 seconds after the stimulation. The ambient illumination of light was kept steadily during measurements to avoid changing pupil size due to pupillary reaction to light.

### Statistical Analyses

Data were statistically analyzed using commercial software (SigmaPlot, version 12.0; HULINKS, Inc.). Comparisons between two groups were made by Student’s *t* tests. The level of statistical significance was set at *P*<0.05. To analyze the degree of association between velocity and maximum amplitude of accommodation, Pearson’s correlation coefficient was calculated.

## Results

### Amplitude of Accommodation

**Figure 4:**
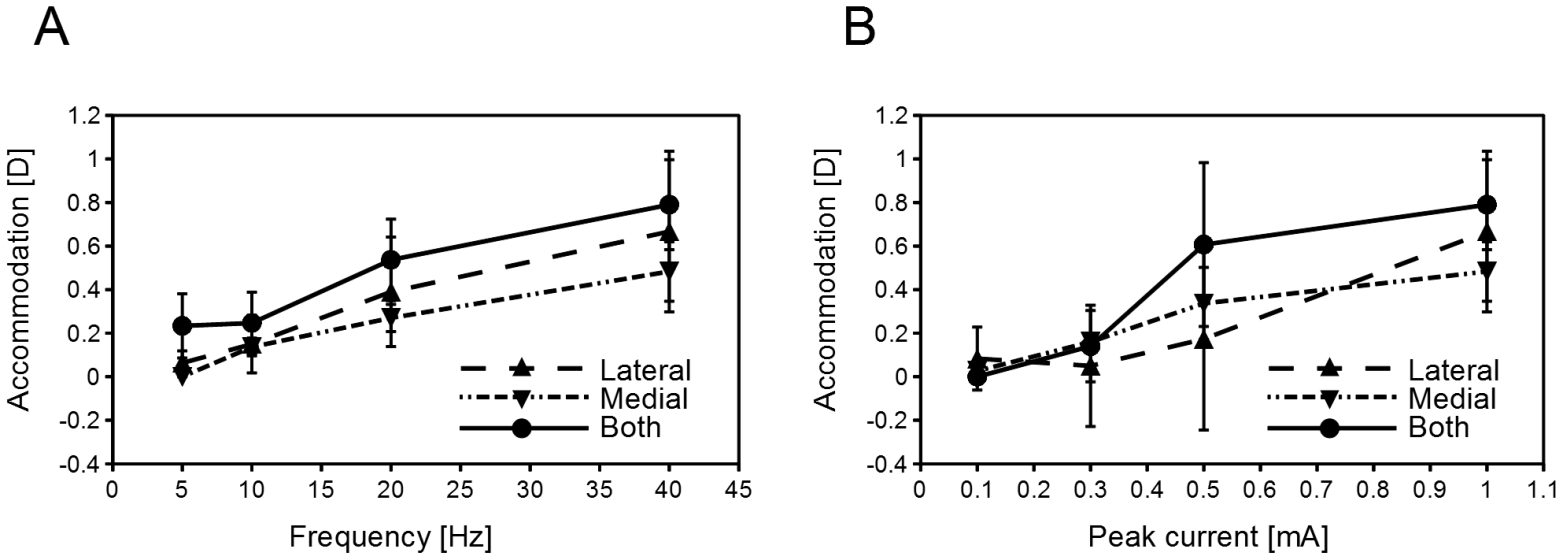
Maximum amplitude of accommodation as a function of the frequency (A) or current (B) of the stimulus. A: The frequency was varied and the current was fixed at 1 mA. B: The current was varied and the frequency was fixed at 40 Hz. One or 2 drops of phenylephrine hydrochloride was instilled prior to the measurements. The maximum amplitude of accommodation is the average of four eyes of four cats (Cat #2, #4, #6, #7). Error bars indicates standard deviations. Monophasic square pulses at a fixed pulse width of 0.5 ms were applied to the lateral, medial, or both branches of the short ciliary nerve.

The average amplitude of accommodation due to stimulation of the medial or the lateral ciliary nerves was 0.64±0.34 D (mean ± standard deviations) by a stimulation of the medial or lateral branch of ciliary nerve with a range of 0.25 to 1.19 D (Table 1). The amplitude of accommodation increased with increasing currents and frequencies of stimulation (Figure 4). However, increasing the currents >1 mA or the frequencies >40 Hz did not increase the amplitude of accommodation significantly. When both branches of the ciliary nerve were stimulated, the accommodative responses were greater than when only one branch was stimulated in 3 of 4 cats.

**Table 1:** Maximum accommodative response.

	Maximum Accommodative Response (Diopter)		
No.	Lateral	Medial	Both
#1	1.08	0.88	
#2	0.25	0.46	0.67
#3	1.19	0.30*	
#4	0.80*	0.36*	1.01*
#5	0.58*	0.20*	
#6	0.95*	0.63*	0.60*
#7	0.18*	0.20*	0.41*

Trains of monophasic square pulses were applied to the lateral, medial or both branches of the short ciliary nerve(1 mA, 40 Hz, 8 sec). The maximum accommodative amplitudes were evaluated by the changes in the refractive power.

*: 1 or 2 drops of Phenylephrine Hydrochloride was instilled.

### Dynamic Accommodative Responses

**Figure 5:**
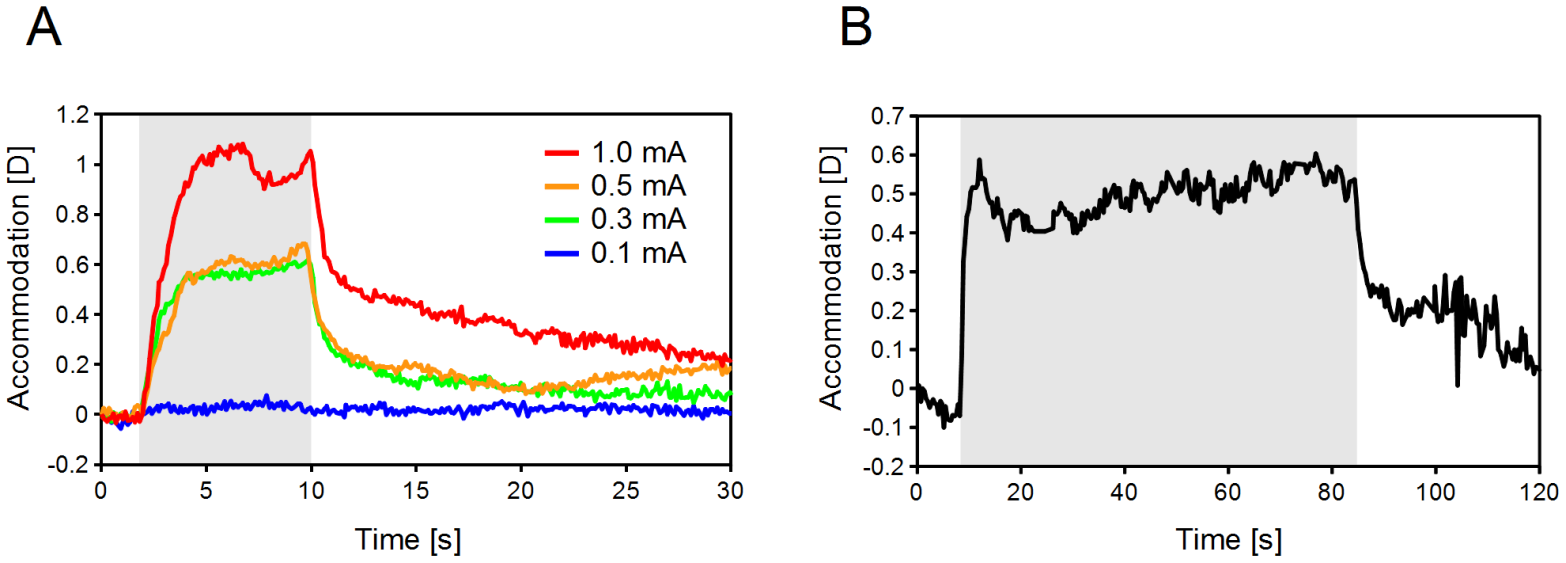
Effect of current and stimulus duration on accommodative responses. A: Typical accommodative responses (Cat #1). Trains of monophasic square pulses of different currents were applied to the lateral branch of the short ciliary nerve. The pulse width and frequency were fixed at 0.5 ms and 40 Hz respectively. B: Efffect of continuous stimulation. A current of 1 mA, frequency of 20 Hz, and duration of pulse train of 75 sec were applied. Shaded areas: Time stimulation was applied.

The accommodative responses were obtained by a sequential recording of the wavefront aberrations. The latency of accommodation was always shorter than the detection limit, <100 ms, of our instrument. The accommodation amplitude continued to change during the 4 seconds after the onset of stimulation (Figure 5A). With longer stimulus durations, the amplitude of accommodation reached and maintained a steady state during the stimulation (Figure 5B). After the stimulation, the accommodation decreased slowly to the original level within 10 sec.

**Figure 6:**
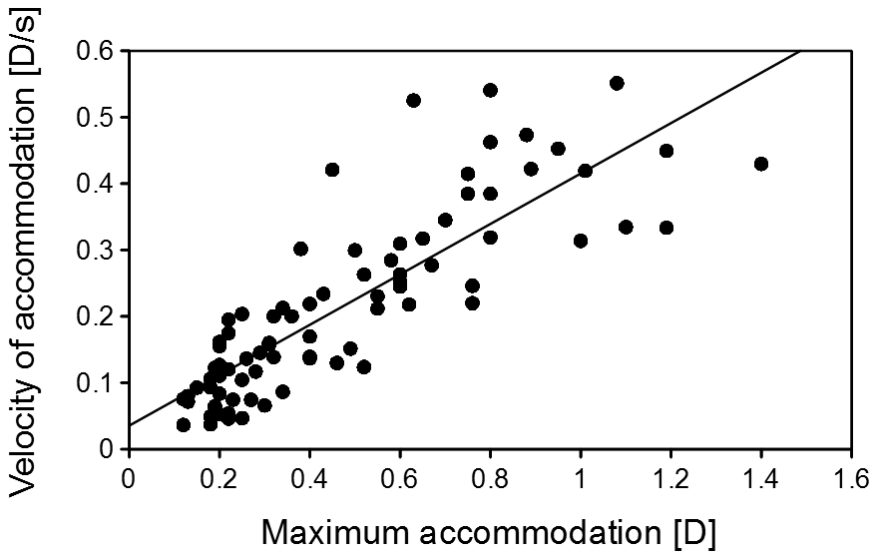
Changes in the velocity of accommodation as a function of maximum accommodation. Stimulus was applied to the lateral or medial or both branches of the ciliary nerve. The data from all seven cats are plotted. Correlations between the velocity of accommodation and maximum accommodation made by Pearson’s correlation (r = 0.839, *P*<0.001).

The velocity of accommodation varied among the trials. A comparison of the velocities of accommodation and the maximum amplitude of accommodation are shown in Figure 6. The velocities increased significantly with increasing maximum accommodation (Pearson’s correlation; r = 0.839, *P*<0.001).

### Dynamic Pupillary Dilation

**Figure 7:**
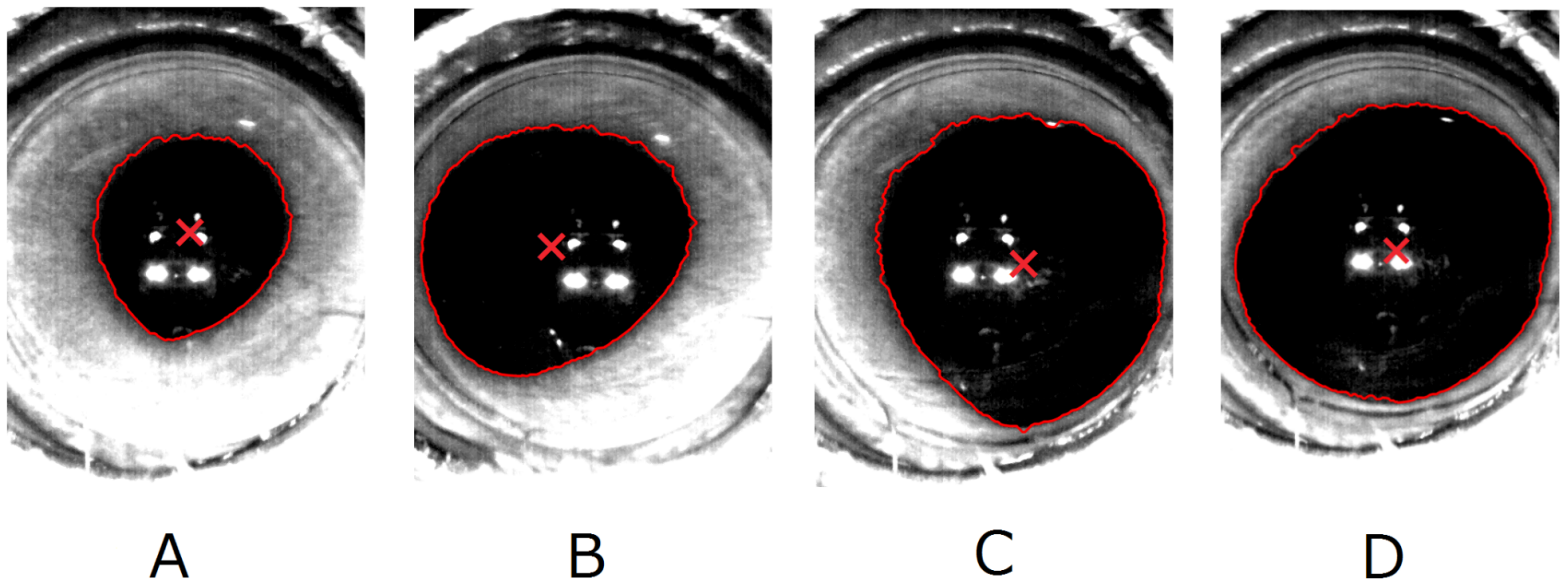
Pupillary images before and after electrical stimulation of the ciliary nerve (Cat #2 right eye). A: Before stimulation. B: Maximum dilation when the lateral branch of the short ciliary nerve was stimulated. C: Maximum dilation when medial branch was stimulated. D: Maximum dilation when both side of branch was simultaneously stimulated. Solid line: Detected contour of the pupil. X: The center of the pupil that is represented as the center of gravity that was calculated from contour data.

The pupil dilated asymmetrically when one branch of the ciliary nerve was stimulated, but if both branches of the ciliary nerve were stimulated, the pupil dilated symmetrically. The pupil never constricted in response to the stimulation parameters used. Representative images of the pupils are shown in Figure 7. The pupillary image before stimulation is shown in Figure 7A. Stimulating the lateral branch (7B) or the medial branch (7C) of the short ciliary nerve produced asymmetric dilation. The pupillary image when both short ciliary nerves were stimulated by the same parameters is shown in Figure 7D.

**Figure 8:**
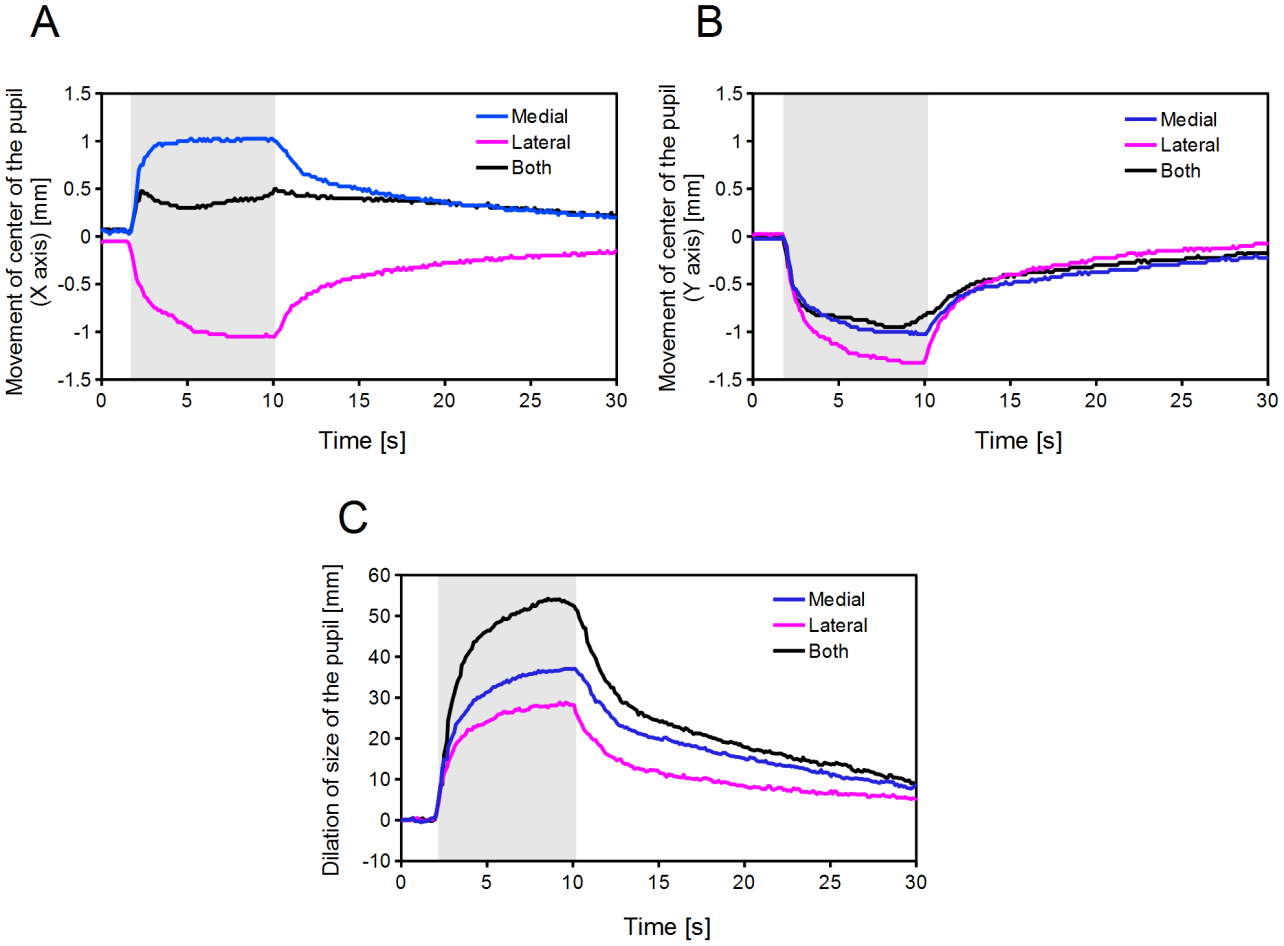
Time course of the changes in the pupillary response to electrical stimulation of one or both branches of the short ciliary nerve (Cat #2). A: Horizontal movement of the center of the pupil. The direction of medial side is represented by positive x-axis, the lateral side is represented by negative x-axis. B: Vertical movement of the center of the pupil. The superior direction is represented by upward dilation, and the inferior direction is represented by downward dilation. C: Changing of the pupil size calculated from the detected contour data. Shaded areas: Stimulation (the pulse train was continuously applied).

The time course of the pupillary dilation is shown in Figure 8. The pupil dilated laterally in the case of lateral branch of the short ciliary nerve was stimulated and the pupil dilated medially in the case of medial branch of the short ciliary nerve was stimulated. The pupil dilated symmetrically when both branches were stimulated (n = 2).

The latencies of the pupillary responses were always shorter than the detection limit (less than 100 ms). After the stimulation, the pupil gradually returned to the original state within several tens of seconds.

### Wavefront Aberrations

**Figure 9:**
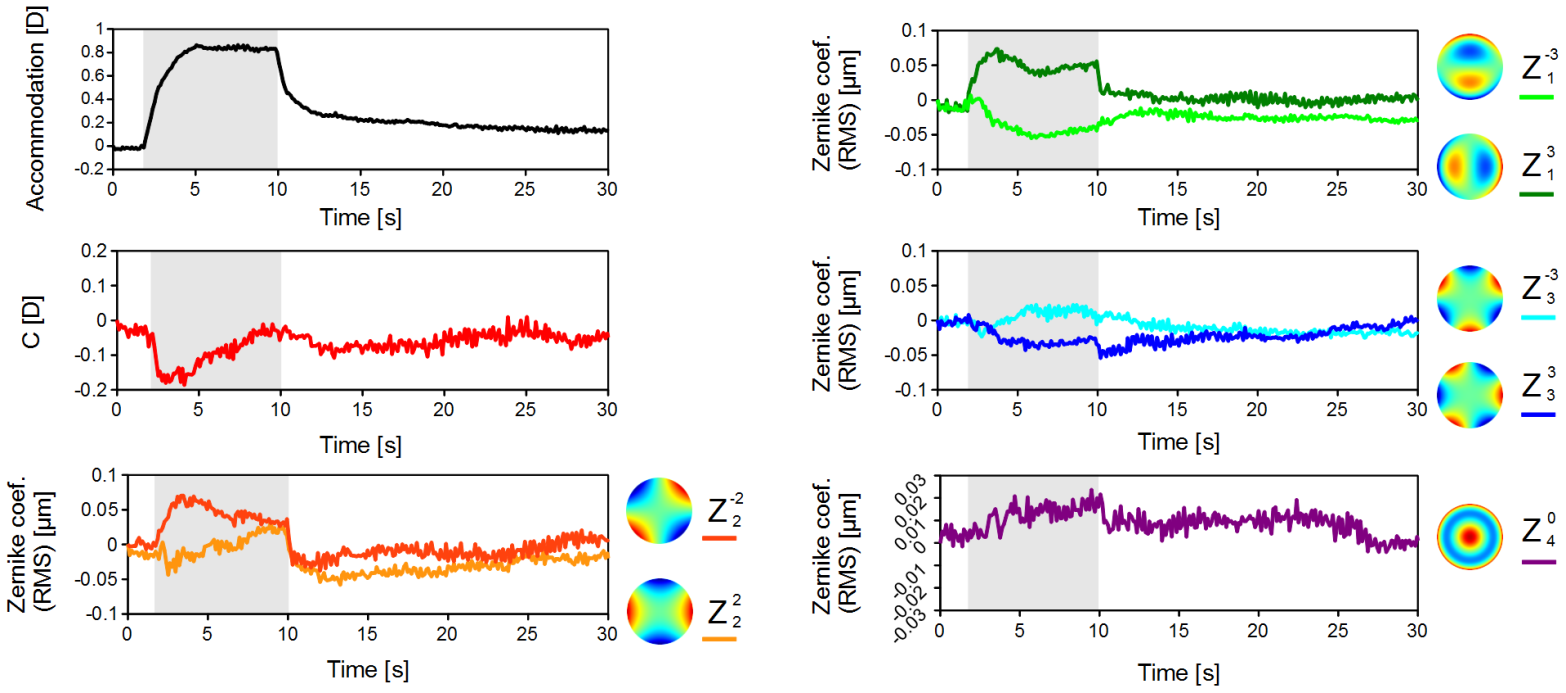
The time course of the changes in the Zernike coefficients and accommodative changes. (Cat #1) S. E.: Spherical Equivalent of refractive change, C: Cylindrical value. All data are the values of the change from the initial state. Shaded areas: Stimulation (the pulse train is continuously applied). The current of 1 mA, the frequency of 40 Hz, the duration of pulse train of 8 seconds were applied to the lateral branch of the short ciliary nerve.

**Figure 10:**
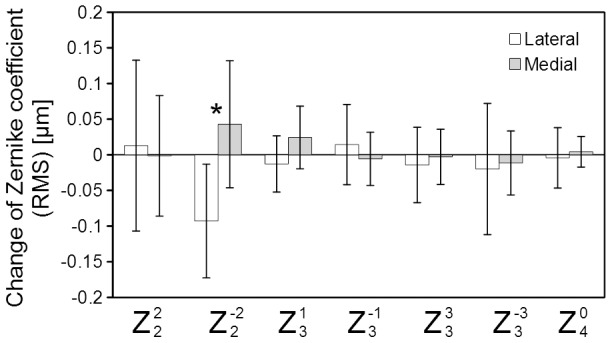
The average change of Zernike coefficients among the seven cats. Error bars represent the standard deviations. A current of 1 mA, frequency of 40 Hz, and duration of pulse train of 8 seconds were applied. Comparisons between the two cases (lateral or medial branch of short ciliary nerve stimulation) were made by Student’s *t* tests. The level of statistical significance was set as P<0.05. (*: P<0.05).

The wavefront aberrations changed with accommodation. A typical example of the time course of the Zernike coefficients and accommodative responses are shown in Figure 9. The time courses of changes in Zernike terms were similar to those of the accommodative responses. The averages and standard deviations of the Zernike coefficients in the seven cats are shown in Figure 10. Zernike coefficients up to 6th order can be calculated with our software. However, the 4 to 6th order of the Zernike terms except for spherical aberration term (Z^0^_4_) are not shown in the results because these terms changed only slightly in almost all trials. The changes of the Zernike coefficients were determined by subtracting the maximum value during the stimulation from the pre-stimulation value. The stimulation parameters were fixed with a peak current of 1 mA, frequency of 40 Hz, and pulse width of 0.5 msec.

We compared the findings between stimulating the lateral and medial branches of the short ciliary nerve. Significant statistical differences were found in the oblique astigmatism term. However, significant statistical differences were not found for all of the other Zernike terms.

## Discussion

Our results showed that stimulation of the short ciliary nerve leads to simultaneous changes in the accommodation, pupillary diameter, and wavefront aberrations. The pupil was never constricted during the stimulation of the branches of short ciliary nerve. Lens accommodation is under the control of the parasympathetic system, while the pupil is under the control of the sympathetic system. Our findings indicate that the nerve bundles which were stimulated contained both sympathetic and parasympathetic nerve fibers. In fact, Kuchiiwa et al. showed in their anatomical studies that the short and long ciliary nerves fuse close to the eye in both the medial and lateral divisions of the short ciliary nerve [28], [29]. The other possible cause of these responses is due to stimulating the sensory nerve in the short ciliary nerve.

The amplitude of accommodation increased with an increase in the frequency and the current of stimulation (Figure 4). The maximum amplitude of accommodation was 1.19 diopters, and increasing the currents >1.0 mA and frequencies >40 Hz did not increase the amplitude of accommodation.

In earlier studies, the maximum accommodation in cats was around 2 diopters when the ciliary ganglion was stimulated [1]–[3]. In addition, the near point of physiological accommodation in cats was estimated to be between 25 to 36 cm or 2.8 to 4.0 D [30]. The maximum amplitude of accommodation under our conditions was less than these values. This disagreement might be caused by the contact between hook shaped electrodes and nerve bundle, and the hook electrodes placed on the nerve bundle might have stimulated only a part of it.

The latencies of accommodation were always less than the detection limit of our recording system (Figure 5). Earlier studies showed that the latencies were >200 ms in the case of ciliary ganglion or midbrain stimulation in cats [27], [31], [32]. This discrepancy might be caused by a difference of the stimulation sites.

The maximum velocity of accommodation was 0.6 D/s in this study taking 4 seconds to reach the peak of accommodation (Figure 4). In addition, we found that the velocity of accommodation was significantly correlated with the amplitude of accommodation (Figure 6). This is in good agreement with previous studies in humans and rhesus monkeys [7], [8], [33]. The latencies might depend on the region stimulated, and the velocity of accommodation may depend on the mechanical properties of the ciliary body and the crystalline lens.

The pupil was asymmetrically dilated when one branch of the ciliary nerve was stimulated (Figures 7 and 8). This suggests that each branch innervates localized areas of the dilator muscle of the pupil. Asymmetric pupillary dilation indicates that the ciliary muscle may also be asymmetrically constricted by stimulation of the short ciliary nerve on one side. If ciliary muscle constricted asymmetrically, asymmetric terms of wavefront aberrations should be changed.

In all trials, the pupil was dilated or was kept stable in size, but never constricted to any of the stimulation parameters. This might be explained by a concurrent stimulation of both parasympathetic and sympathetic nerve fibers. In cats, the sympathetic fibers are reported to be incorporated in the short ciliary nerves after the nerves have been joined by the long ciliary nerves somewhere between the ciliary ganglion and the eye [34]. At the area of the branch of short ciliary nerve which was stimulated in this study, about 5 mm from sclera, the parasympathetic and sympathetic fibers might be mixed. Our results indicated that the stimulation of mixed sympathetic and parasympathetic fibers will cause a dilation. The discharge rate of the mixed ciliary nerve is increased with spontaneous pupillary dilation in cats [35].

The wavefront aberrations change with accommodation in humans, and the spherical aberration (Z^0^_4_) shows the greatest change among all the Zernike terms [12]. The changes in the astigmatism and the coma terms were smaller than that for spherical aberration. In contrast, the changes in the spherical aberration was smaller than for the astigmatism and coma terms (Figures 9 and 10). These discrepancies may be due to differences in the shape of the crystalline lens between humans and cats. The crystalline lens of cats is more spherical in shape than that of humans.

The differences in the changes in the oblique astigmatism term (Z^−2^_2_) between stimulation of the lateral or the medial branch of the short ciliary nerve were significant (Figure 10). This suggests that an asymmetrical constriction of ciliary muscle is induced by unilateral ciliary nerve stimulation, which may induce the deformation of crystalline lens. In patients with Adie’s syndrome, astigmatism is reportedly induced after accommodation [23]. Our findings in cats support the hypothesis that a segmental constriction of ciliary muscle occurs when patients with Adie’s syndrome accommodates which eventually causes the increase of asymmetrical astigmatism.

Further study is necessary to confirm the localized innervation of the short ciliary nerve that lead to the asymmetric contraction of the ciliary muscle. It is also necessary to make a computer simulation if the asymmetric movement of ciliary muscle induces the deformation of crystalline lens.

In conclusion, we measured the dynamic change of the wavefront aberrations, pupillary size and shape, and accommodation simultaneously and serially with a custom-built compact wavefront aberrometer. The asymmetric pupillary dilation and asymmetrical changes of the wavefront aberrations with accommodation elicited by electrical stimulation on one branch of the ciliary nerve suggest that the branch of ciliary nerve innervates localized regions of the dilator of pupil and ciliary muscle.
